# Effectiveness of human, camel, bovine and sheep lactoferrin on the hepatitis C virus cellular infectivity: comparison study

**DOI:** 10.1186/1743-422X-10-199

**Published:** 2013-06-19

**Authors:** Esmail M EL-Fakharany, Lourdes Sánchez, Hussein A Al-Mehdar, Elrashdy M Redwan

**Affiliations:** 1 Therapeutic and Protective Protein Laboratory, Protein Research Department, Genetic Engineering and Biotechnology Research Institute, City for Scientific Research and Technology Applications, New Borg EL-Arab, Alexandria 21394, Egypt; 2 Tecnología de los Alimentos, Facultad de Veterinaria, Universidad de Zaragoza, Miguel Servet 177, Zaragoza 50013, Spain; 3 Biological Sciences Department, Faculty of Science, King Abdulaziz University, P.O. Box 80203, Jeddah 21589, Kingdom of Saudi Arabia

**Keywords:** Comparison, Different lactoferrin, Hepatitis C virus, Anti-infectivity

## Abstract

**Purpose:**

The prevalence of HCV infection has increased during recent years and the incidence reach 3% of the world's population, and in some countries like Egypt, may around 20%. The developments of effective and preventive agents are critical to control the current public health burden imposed by HCV infection. Lactoferrin in general and camel lactoferrin specifically has been shown to have a compatitive anti-viral activity against hepatitis C virus (HCV). The purpose of this study was to examine and compare the anti-infectivity of native human, camel, bovine and sheep lactoferrin on continuous of HCV infection in HepG2 cells.

**Material and methods:**

Used Lfs were purified by Mono S 5/50 GL column and Superdex 200 5/150 column. The purified Lfs were evaluated in two ways; 1. the pre-infected cells were treated with the Lfs to inhibit intracellular replication at different concentrations and time intervals, 2. Lfs were directly incubated with the virus molecules then used to cells infection. The antiviral activity of the Lfs were determined using three techniques; 1. RT-nested PCR, 2. Real-time PCR and 3. Flowcytometric.

**Results:**

Human, camel, bovine and sheep lactoferrin could prevent the HCV entry into HepG2 cells by direct interaction with the virus instead of causing significant changes in the target cells. They were also able to inhibit virus amplification in HCV infected HepG2 cells. The highest anti-infectivity was demonstrated by the camel lactoferrin.

**Conclusion:**

cLf has inhibitory effect on HCV (genotype 4a) higher than human, bovine and sheep lactoferrin.

## Background

The hepatitis C virus (HCV) is a major health problem in the world and a leading cause of chronic liver disease [1] and with an estimated about 180 million people is infected worldwide. HCV is currently the most significant public health problem in Egypt with an infection prevalence of up to 20%, this is ten times greater than any other country in the world and the highest prevalence of HCV genotype 4, which is responsible for 90% of infections, with a predominance of subtype 4a (55%) [2-4]. Studies suggest that mortality related to HCV infection will increase over in the next two decades [5]. A protective vaccine against HCV does not exist till now, and current standard treatment for chronic HCV infection is interferon α alone or in combination with ribavirin. This treatment of HCV is costly, requires more time (12–72 weeks) to complete, and has serious adverse effects of ribavirin is hemolytic anemia that may require dose reduction, low efficiency and discontinuation of treatment. The developing new treatment against HCV has been hampered by difficulties in replicating the virus in cell culture and the lack of suitable animal models.

Lactoferrin (Lf) is an 80 kDa multifunctional glycoprotein belonging to the transferrin family. Lf is primarily present in milk, and is also found in other biological fluids, such as saliva, tears, bile and pancreatic juice [6]. It has been widely documented that Lf displays antimicrobial activity against many different pathogenic agents. This activity was attributed to its ability, to bind iron with a high affinity and unlike transferrin, retain its bound iron under acidic conditions. Also Lf is considered to be a part of the innate immune system and takes part in specific immune reactions, but in an indirect way [7]. The antiviral activity of Lf is directed against a broad spectrum of viruses, including both RNA- and DNA-viruses, enveloped as well as naked viruses. Lf saturated with iron ions has been shown to exhibit antiviral activity against HSV-1 and HSV-2 [8,9]. Lf also exhibit antiviral properties against HCV. Ikeda et al. [10] have shown that Lf is able to prevent the infection of HCV in the cultured human hepatocytes cell line PH5CH8. Since pre-incubation of Lf and HCV was required to prevent infection of the cells it was hypothesized that the inhibition happens through a direct interaction between the virus and Lf. This idea is supported by the fact that pre-incubation of the PH5CH8 cells with Lf had no inhibitory effect on HCV, indicating that the antiviral activity of Lf against HCV was not due to the interaction of Lf with the cells.

Bovine and human Lfs are able to bind to the HCV envelope proteins E1 and E2 [11]. This binding inhibits any possible interaction of the virus with its cellular receptors. In all cases studied, it appears that Lf exerts its antiviral activity at an early phase in the infection process. Similar results have recently been reported for camel lactoferrin (cLf), demonstrating complete inhibition of virus entry when cLf and HCV were preincubated together, while Lf pre-incubation with human leukocytes [12], HepG2 cells [13] and Huh7.5 cells [14,15] prior to HCV infection had no effect on viral entry. Both full length of native and recombinant clf were shown similar results [14]. The enzymatic prepared of native N- lobe, C-lobe and recombinant N-lobe has been shown a similar effect against HCV cellular infectivity [14]. Several viral pathogens has been shown to use host cell surface HS as an attachment receptor during the infection process and Lf also binds HS [16]. The results have shown that HS at the cell surface is important for Lf to exert antiviral activity [17,18]. The objective of this study was to examine and comparison the potential inhibitory effects of human, camel, bovine and sheep lactoferrin on HCV entry and amplification in HepG2 cells.

## Results

### Purification of native Lfs

**Figure 1:**
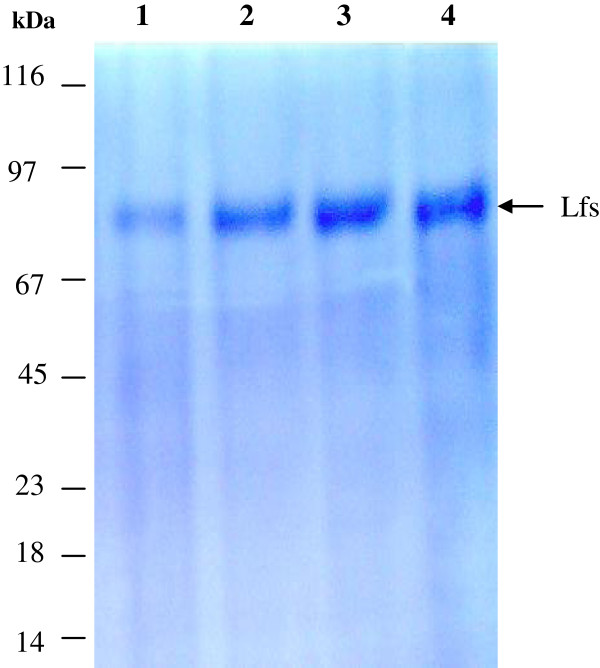
**12% SDS-PAGE analysis of different types of purified Lfs (hLf, cLf, bLf and sLf).** Lanes 1–4 purified human, camel, bovine and sheep lactoferrin, respectively.

Human, Camel, Bovine and Sheep lactoferrins were isolated and purified in two steps with cation exchange resin (Mono S 5/50 GL column) and gel filtration chromatography (Superdex 200 5/150 column). The skimmed milk by was loaded to Mono S 5/50 GL column and the Lfs were eluted at a salt strength of 0.0-1.0 M NaCl. The peak containing Lf was concentrated and applied into Superdex 200 5/150 column. Only one band was visualized on 12% SDS-PAGE of the protein for all Lfs. The electrophoretic analysis of the protein eluted revealed that the presented a single protein band corresponding to about 80 KDa (Figure 1) without clear differences in the molecular weight of all lactoferrins.

### Cytotoxic effect of hLf, cLf, bLf and sLf

To exclude the possibility that the elimination of the HCV was caused by the reduced viability of the cells, the cytotoxic effects of Lfs on the cells were investigated. PBMCs and HepG2 cells were treated with each protein at concentrations of 0.5 and 1.0 mg/ml for 4 days. Cell viability was compared with that of untreated PBMCs and HepG2 cells. The results showed that all used Lfs had no adverse effects on the viability of PBMCs at concentrations of 0.5 or 1.0 mg/ml after incubation for 4 days as shown in Table 1. While in case of HepG2 cells, the viability was reduced to around 90% only after 4 days of incubation at concentrations of 0.5 or 1.0 mg/ml for all used Lfs as shown in Table 1.

**Table 1:** Cell viability of lactoferrin forms by MTT method

	**HepG2 Cells**		**PBMCs**	
	**0.5 mg/ml**	**1.0 mg/ml**	**0.5 mg/ml**	**1.0 mg/ml**
Control	100	100	100	100
Camel Lf	92	89	100	100
Human Lf	95	90	100	100
Bovine Lf	94	88	100	100
Sheep Lf	91	88	100	100

### Inhibition Potential of hLf, cLf, bLf and sLf

**Figure 2:**
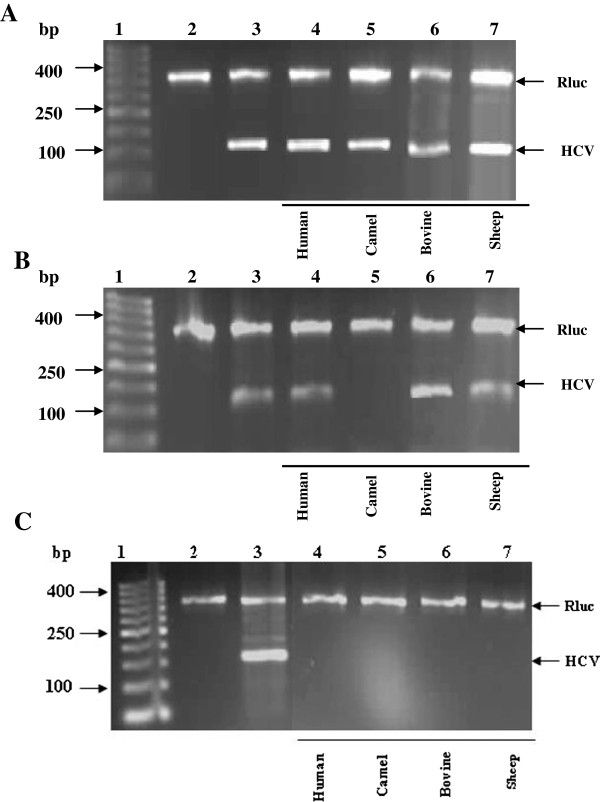
**Prevention of HCV entry into HepG2 cells by different types of Lfs (hLf, bLf, cLf and sLf).** Lane 1, DNA ladder; lane 2, negative control (HepG2 cells served as negative control); lane 3, positive control (infected HepG2 cells with HCV served as positive control); lanes 4–7, comparison among different Lfs at concentration of 0.25 mg/ml (**A**), 0.5 mg/ml (**B**) and 1.0 mg/ml (**C**), as indicated under the gel graph. Rluc served as internal control.

All the Lfs used were demonstrated ability to completely inhibit HCV particles entry into HepG2 cells at concentration of 1.0 mg/ml. HepG2 cells (10^5^) were cultured in duplicate as described in materials and methods section. The cells were inoculated with HCV infected sera pretreated with each Lf alone at concentrations of 0.25, 0.5 and 1.0 mg/ml for 60 min and cultured for seven days. The RT-nested PCR amplified the 174 bp region at the 5′ of HCV non coding sequence in comparison to the positive and negative control. The result revealed that the clf, was able to completely inhibit the HCV entry into HepG2 cells at concentrations of 0.5 and 1.0 mg/ml. While the results showed that the hLf, bLf and sLf have a similar ability of cLf to completely inhibit the HCV entry into cells at concentration of 1.0 mg/ml only, but at concentrations 0.25 mg/ml for all used Lfs were failed to prevent or block HCV particles from entry into HepG2 cells as shown in Figure 2.

## Effect of hLf, cLf, bLf and sLf on intracellular replication of HCV

### Using RT-nested-PCR

**Figure 3:**
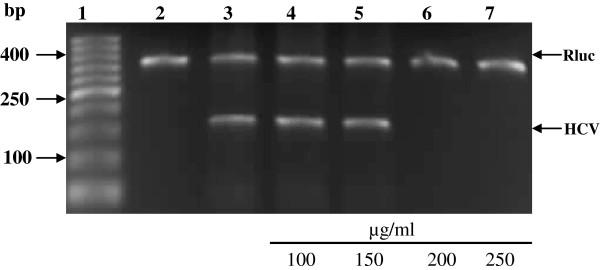
**Inhibition of HCV amplification in infected HepG2 cells by camel lactoferrin at different concentrations.** Lane 1, DNA ladder; lane 2, negative control (HepG2 cells served as negative control); lane 3, positive control (infected HepG2 cells with HCV served as positive control); lane 4–7, comparison among different concentrations of cLf. Rluc served as internal control.

**Figure 4:**
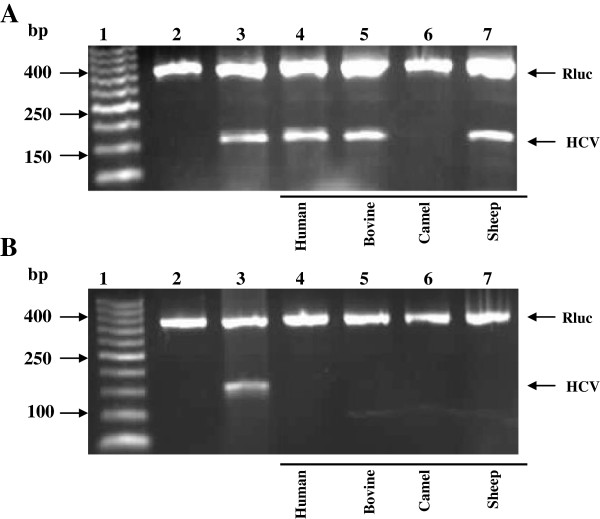
**Inhibition of HCV amplification in infected HepG2 cells by different types of Lfs.** Lane 1, DNA ladder; lane 2, negative control (HepG2 cells served as negative control); lane 3, positive control (infected HepG2 cells with HCV served as positive control); lane 4–7, comparison among different types of LFs at concentration of 0.25 mg/ml **(A)** and 0.5 mg/ml **(B)**. Rluc served as internal control.

In order to determine the inhibitory effects of Lf on HCV replication in infected HepG2 cells, Lf treatment was performed at different concentrations. Results showed that cLf, hLf, bLf and sLf effectively inhibit HCV replication in infected HepG2 cells. hLf, bLf and sLf at concentrations of 0.25 and 0.5 mg/ml and cLf at concentrations of 100, 150, 200, 250 and 500 μg/ml were investigated for their *in vitro* ability to inhibit the viral replication inside the infected HepG2 cells. Camel Lf has the ability to inhibit HCV replication at concentrations starts from 200, 250 and 500 μg/ml after four days of treatment, but cLf at concentrations of 100 and 150 μg/ml was failed to block the HCV replication inside the infected cells as shown in Figure 3. However, human, bovine and sheep Lfs were able to completely inhibit the replication of HCV at concentration starts from 0.5 mg/ml, while at concentration of 0.25 mg/ml, those proteins were failed to prevent HCV replication inside infected HepG2 cells as shown in Figure 4.

### Using real time PCR

The results were indicated that the camel lactoferrin at all concentrations used was able to prevent HCV particles from replication inside HepG2 cells completely as shown in Table 2. The activity of bovine lactoferrin became 100% at concentration 0.5 and 0.75 mg/ml and drastically declined to become 7.28% at concentration of 0.25 mg/ml. However, sheep Lf was able to complete inhibition of HCV replication inside infected HepG2 cells with relative activity 100% at concentrations of 0.5 and 0.75 mg/ml and the activity was decreased to become 16.73% at concentration of 0.25 mg/ml (Table 2). While the activity of human lactoferrin on HCV replication inside infected HepG2 cells decreased from 100% at concentrations of 0. 5 and 0.75 mg/ml to become 24.48% at concentration of 0.25 mg/ml as shown in Table 2.

**Table 2:** Quantitation in vitro comparison among different types of LFs (human, bovine, camel and sheep) activity against HCV-infected HepG2 cells

**Protein**	**Protein conc.(mg/ml)**	**Calc. conc. (IU/ml)**	**Relative activity (%)**
control	Positive	100410	0.0
	Negative	0.0	100
Human LF	0.25	75820	24.48
	0.5	0	100
	0.75	0	100
Bovine LF	0.25	93100	7.28
	0.5	0	100
	0.75	0	100
Camel LF	0.25	0	100
	0.5	0	100
	0.75	0	100
Sheep LF	0.25	83610	16.73
	0.5	0	100
	0.75	0	100

### Using flow cytometry

**Figure 5:**
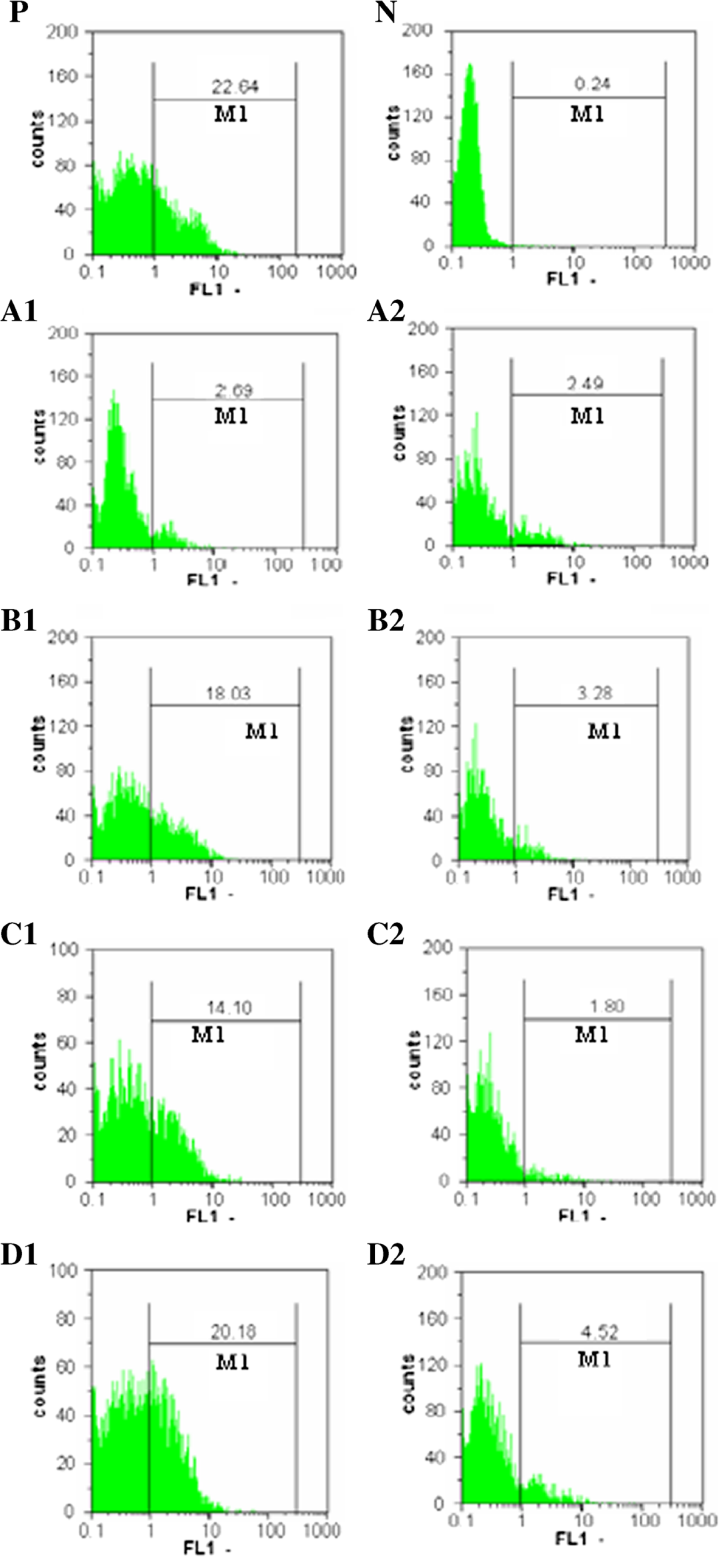
**Histograms from gated cells obtained from flowcytometric analysis of HCV-infected HepG2 cells and treated with Lf forms.** Infected HepG2 cells with HCV served as positive control (P) and HepG2 cells served as negative control (N). Cells were stained intracellularly with anti-rabbit antibody conjugated with FITC. Effect of camel, bovine, human and sheep Lf (**A**, **B**, **C** and **D**) at concentrations of 0.20 and 0.5 mg/ml (1 and 2), respectively. M1 is positive stained cells.

The obtained results using flowcytometery were confirmative for the above results through evaluation of the effect of the lactoferrin forms on intracellular burden and may tracing the HCV by the indirect intracellular immunostaining of HCV antigens with the flowcytometry. Camel Lf could inhibit the replication of HCV at concentrations of 0.20 and 0.5 mg/ml after four days of treatment (Figure 5). However, human, bovine and sheep Lfs were accomplished their HCV replication inhibition at 0.5 mg/ml concentration as detected high fluorescence signal in FACS scan profile (Figure 5).

## Discussion

Lactoferrin is an iron binding protein, which a member of the transferrin family present mainly in breast milk and in lower extent in bile, tears and in other exocrine secretions. However, Lf is also found in plasma derived from predominantly neutrophil secondary granules, release of which increases during inflammation. Breast milk contains several components with antimicrobial activity. The most one with antiviral activity detected in milk can be ascribed to Lf [15,19,20]. The antiviral activity of Lf is directed against a broad spectrum of viruses, including both RNA- and DNA-viruses. Because of the importance of HCV as a human pathogen and the lack of an effective treatment or protective vaccine [21,22], there is a crucial need to screening new compounds having anti-HCV activity with lower side effects. Previously we have shown that camel lactoferrin inhibits HCV genotype 4 from entry to PBMC, HepG2 and HepG2 cells and cLf has also been shown to inhibit the HCV G4 replication inside infected cells [12-15,20]. In the current study, we were tested and compared the inhibitory effect of hLf, bLf, cLf and sLf against HCV G4.

This is the first study showing that the entry of HCV G4 to HepG2 cells and the replication of the virus inside it are prevented by hLf, cLf, bLf and sLf. The results demonstrated that the cLf displayed its effective inhibition against HCV entry as well as replication in infected HepG2 cells more than hLf, bLf and sLf. Current study indicated that the cLf inhibit the HCV entry into HepG2 cells at concentrations of 0.5 and 1.0 mg/ml, while hLf, bLf and sLf have the same ability of cLf to inhibit the HCV entry into cells but at concentration of 1.0 mg/ml, whereas they were failed to inhibit the entry of the virus into cells at concentration of 0.25 mg/ml. However, at all concentrations the cLf was able to prevent HCV particles from replication inside the HepG2 cells, The activity of hLf decreased from 100% at concentrations of 0.5 and 0.75 mg/ml to became 24.48% at concentration of 0.25 mg/ml, and bLf activity was decreased from 100% at concentration 0.5 and 0.75 mg/ml to became 7.28% at concentration of 0.25 mg/ml. Whereas, sLf was able to inhibit HCV replication inside infected cells with relative activity of 100% at concentrations of 0.5 and 0.75 mg/ml and its activity was 16.73% at concentration of 0.25 mg/ml.

The HCV inhibition potential was simultaneously analyzed at viral RNA level (by RT-nested-PCR, real time PCR) and furthermore, using the highly sensitive intracellular staining for detection of the specific viral proteome. It seems that the flow cytomtery could exactly show the dose dependent decrease of the intracellular viral signals, and confirm the RNA viral load [23] through the ability to count the rate of infected cells directly. It precisely, when a typical forward and side scatter was classified to differentiate stained and non-stained cells or specific and non-specific immunofluorescence signals, was clear correlations between the flow cytometry and RT-PCR/real-time PCR. This approach appeared to be useful and dependable for anti-HCV agent discovery and/or their follow up [12,24]. Our findings are consistent with several previous studies, those using human and bovine lactoferrin to inhibit HCV (genotype1) entry into the PH5CH8 cell-line [25,26].

Many viruses are inhibited by Lf, which exhibits its antiviral activity at early stage of infection, most probably through preventing virus entry by interacting with the viral attachment receptor heparan sulfate [17,18,27,28]. However, the blocking of viral binding receptors on the cell surface cannot fully explain the antiviral activity of Lf [13,29]. Also many studies were analyzed the effect of Lf on picornavirus infection with different results. Marchetti et al. [30] demonstrated that bLf and hLf inhibited the early phases of poliovirus infection, whereas they were ineffective when added after the viral adsorption step. Successively, it was reported that hLf did not affect rhinovirus replication [31], while bLf exerted an inhibitory effect on enterovirus 71 attachment to target cells [32]. Camel Lf has three characteristics which make it unique over Lf of other species: 1) some critical residues such as Pro418, Leu423, Lys433, Gln651, Gly629, Lys637, Arg652, and Pro592 related to domain movement are different in cLf from those found in other Lf species, indicating of specific structural-related differences, 2) cLf loses 50% of its iron contents at pH 6.5 and the remaining 50% is lost at acidic condition (pH 4.0-2.0). The N-lobe lost iron at acidic pH less than 4.0, whereas the C-lobe lost iron at pH 6.5, which indicating a difference in the iron release mechanism from the two lobes, 3) the entirely difference in predicated glycosylation sites in cLf from other species. These data demonstrate that the cLf acts as half lactoferrin “iron binding protein” and half transferring “irontransporter protein”, unlike other lactoferrins and transferrins [33]. A recent study showed that the therapy with bovine Lf lead to lipid peroxidation inhibition [34]. In accordance, the camel Lf maintains a dual function; 1) it inhibits lipid peroxidation and 2) it regulates the hepatic iron content through its ability to bind and transport the iron at acidic and basic pHs.

## Conclusions

In Conclusion, we demonstrated, for the first time, that the camel lactoferrin has inhibitory activity on HCV (genotype 4a) double folds higher than human, bovine and sheep lactoferrin. However, the question still needs further analysis, why the camel lactoferrin has this superiority? Is it structure dependent, i.e. protein and/or carbohydrates wise?

## Materials and methods

### Processing of milk and lactoferrin purification

Milk from camels (*Camelus dromedarius*) was purchased from ALKHIR camel farm (Matrouh, Egypt) and transferred frozen to our laboratory, Bovine and sheep milk were purchase from local market and human was obtained from 10 healthy feeding mothers. A solution of sodium azide (0.2%) containing 5 mM EDTA and 5 mM PMSF was added to the milk before defatting by centrifugation at 1000 × g for 30 min at 4°C. The pH of skimmed milk was decreased to 4.2 with 1 M HCl to precipitate the casein [13,20]. Skimmed milk was used for Lf purification and diluted with 50 mM Tris–HCl, pH 8.0 then samples containing 100 mg protein was applied to Mono S 5/50 GL column (5 × 50 mm, GE Health care, Sweden) previously equilibrated with 50 mM tris HCl, pH 8.0 and column was washed with same equilibrated buffer to remove impurities. The elution was carried out with 50 mM tris HCl, pH 8.0 and gradient from 0.0 to 1.0 M NaCl at flow rate of 1.0 ml/min and fraction size of 1.0 ml/fraction using AKTA prime plus FPLC (GE Health care, Sweden). The fractions containing Lf were collected and concentrated by amicon ultrafiltration cell (Amicon 8200 Pmax 75 psi, 5.3 Kg/cm2 using 50 kDa MWCO Amicon filter membrane (Millipore, Billerica, USA), then samples containing 0.8 mg protein was applied to Superdex 200 5/150 column (5 × 150 mm, GE Health care, Sweden) previously equilibrated with 50 mM tris HCl, pH 8.0. Elution of all Lfs was carried out with same equilibrated buffer at flow rate of 0.3 ml/min and fraction size of 0.5 ml/fraction. The fractions containing Lf were concentrated by amicon ultrafiltration cell using 50 kDa MWCO Amicon filter membrane (Millipore, Billerica, USA). The Lfs purity was confirmed by SDS-PAGE [35].

### Protein and endotoxin determination

Protein was determined either by measuring the absorbance at 280 nm or by the method of Bradford [36] using bovine serum albumin as a standard protein. The endotoxin level of the purified lactoferrin was checked [37] to avoid its pyrogenic effects on the cell-culture system. All lactoferrin batches used were free of endotoxin (data not shown).

### Infected serum samples

For all infection experiments, PCR-HCV positive serum samples of genotype 4 from Egyptian patient “A.R.” (After approval from our Genetic Engineering and Biotechnology Research Institutes (GEBRI) institutes ethics committee) were used as previously described by Redwan and Tabll [12-15,38,39]. Written informed consent was obtained from the patient for the publication of this report and any accompanying images.

### Cytotoxic effect of lactoferrin forms

The cytotoxicity of the purified Lfs on human separated PBMCs and HepG2 was examined by the 3-(4, 5-dimethylthiazol-2-yl)-2, 5-diphenyltetrazolium bromide (MTT) test [38,40,41]. In brief, about 10^4^ PBMCs and HepG2 cells in 200 μl complete media were plated in 96-well microtiter plates and cultured for overnight at 37°C before treatment with Lf forms, then the medium was refreshed with new DMEM supplemented medium containing 1.0 mg/ml or 0.5 mg/ml of protein. The cells were incubated for four days at 37°C. After incubation, the cells were washed 3 times with PBS, and 200 μl MTT solution (0.5 mg/ml in PBS) was added to each well. After incubation for 3–5 h at 37°C, 5% CO2, the medium was discarded and the wells were dried. Formazan crystals were resuspended in 200 μl dimethyl sulfoxide, followed by shaking for 5 min to thoroughly mix the formazan into the solvent. The optical density was read at 570 nm. The relative cell viability (%) compared to control wells containing cells without adding Lf was calculated using the following formula: (A) test/ (A) control × 100%.

### *In vitro* comparison of neutralizing efficacy of different forms of lactoferrin

#### Inhibition potential of the lactoferrin forms

To examine the interaction of hLf, cLf, bLf and sLf with HCV, 1 ml of infected serum and lactoferrin forms (final concentration at 0.25, 0.5 and 1.0 mg/ml) was pre-incubated with DMEM media containing 2% HCV infected serum for 1 h at 4°C, and then the mixture of HCV and hLf, cLf, bLf or sLf was added to HepG2 (1.0 × 10^5^) cells cultured in 24-well microtiter plate, and incubated for 90 min at 37°C, 5% CO2 and 88% humidity. The cells were washed three times with 1 ml of PBS and further incubated for 7 days at 37°C, 5% CO2 and 88% humidity. (Positive HepG2 (1.0 × 10^5^) cells were infected with HCV and negative HepG2 (1.0 × 10^5^) cells only without infection) control cultures were included. The cells were washed three times from debris and dead cells by using DMEM supplemented media or 1 × PBS, followed by total RNA extraction [12-15,38].

#### Effect evaluation of the different lactoferrin on hepatitis C virus intracellular replication

HepG2 cells were washed twice in DMEM supplemented media. The cells were suspended at 1.0 × 10^5^ cells/ml in DMEM culture media (DMEM supplemented media, 10% fetal bovine serum (FBS); 100 U of penicillin and 100 μg streptomycin). The cells were left to adhere on 24-well plates for 24 h at 37°C, 5% CO2 and 88% humidity, then infected with HCV-infected serum in DMEM media and incubated for 24 h at 37°C, 5% CO2 and 88% humidity. The purified cLf, was added at concentrations 100, 150, 200, 250 and 500 μg/ml, while purified hLf, bLf and sLf were added at concentrations of 0.25 and 0.5 mg/ml. Positive HepG2 (1.0 × 10^5^) cells were infected with HCV and negative HepG2 (1.0 × 10^5^) cells only without infection control cultures were included. The cells were incubated for four days at 37°C, 5% CO2 and 88% humidity. After incubation the cells were washed three times from debris and dead cells by using DMEM supplemented media, then the cells were tested by RT-PCR and real time PCR.

### Isolation and extraction of RNA from HepG2 cells

RNA was isolated from HepG2 cells as previously described [14]. Briefly, cells were precipitated by centrifugation at 1200 rpm for 5 min at 4°C and washed three times with PBS or basal media to remove adherent viral particles before lysis in 4 mol/l guanidine isothiocyanate containing 25 mM sodium citrate, 0.5% sarcosyl and 100 mM β-mercaptoethanol and 100 μl sodium acetate. The lysed cells were centrifuged at 12000 rpm for 10 min at 4°C. The aqueous layer was collected and mixed with equal volume of isopropanol. After incubation at −20°C overnight, RNA was precipitated by centrifugation at 12000 rpm for 20 min at 4°C and the precipitate RNA was washed twice with 70% ethanol.

### RT-nested-PCR of HCV RNA

Reverse transcription-nested PCR was carried out previously reported [12-15,38,42]. The complimentary DNA (cDNA) and the first PCR reaction of the nested PCR detection system for the HCV RNA was performed in a 50 μl volume single-step reaction using the Ready-To-Go RT-PCR beads (Amersham Pharmacia Biotech, Pis-cataway, NJ, USA), 400 ng of total HepG2 cells RNA, 10 μM of the reverse primer 1CH (for plus strand), 10 μM of the forward primer 2CH (for minus strand) and 10 μM of reverse primer P2. The test was incubated at 42°C for 30 min and denatured at 98°C for 10 min. Amplification of the highly conserved 5′-UTR sequences was done using two rounds of PCR with two pairs of nested primers (Clontech, USA). First round amplification was done in 50 μl reaction mixture, containing 10 μM from each of 2CH forward primer and P2 reverse primer, 0.2 mmol/l from each dNTP, 5 μl from RT reaction mixture as template and 2 U of Taq DNA polymerase (Promega, Madison, USA) in a 1× buffer supplied with the enzyme. The thermal cycling protocol was as follows: 1 min at 94°C, 1 min at 55°C and 1 min at 72°C for 30 cycles. The second round amplification was done similar to the first round, except for use of the nested reverse primer D2 and forward primer F2 at 10 μM each. A fragment of 174 bp was identified in positive samples. Primer sequences were as follows: 1CH: 5′-GGTGCACGGTCTACGAGACCTC-3′, 2CH: 5′-AACTACTGTCTTCACGCAGAA-3′, P2: 5′-TGCTCATGGTGCACGGTCTA-3′, D2: 5′- ACTCGGCTAGCAGTCTCGCG-3′ and F2: 5′-GTGCAG CCTCCAGGACCC-3′. To control false detection of negative-strand HCV RNA and known variations in PCR efficiency, specific control assays and rigorous standardization of the reaction were employed. The amplification process was included a Rulc plasmid as internal control, so the final amplified DNA were electrophoresed through 3% agarose gel and ethidium bromide was used to visualized 174 bp for HCV and 374 bp of Rluc.

### Real time PCR to evaluation of antiviral activity of the clf, recombinant clf, N-lobe, recombinant N-lobe and C-lobe against HCV

We used real time PCR to examine the ability of hLf, clf, bLf and sLf at concentrations of 0.25, 0.5 and 0.75 mg/ml to prevent replication of HCV particles inside HepG2 cells. Briefly, infected HepG2 cells with HCV were treated with those proteins and cultured as described above. After incubation of HepG2 cells at suitable time the cells were washed three times from debris and dead cells by using PBS then HCV RNA was isolated and extracted from HepG2 cells by INSTANT Virus RNA Kit (AJ Roboscreen GmbH). Amplification of HCV RNA in samples and standards is measured by RoboGene HCV RNA Quantification Kit (AJ Roboscreen GmbH) use Rotor-Gene real time PCR machine (Corbett life Science, QIAGEN Company, model number R0708103) and report generated by Rotor-Gene Q Series Software 1.7 (Build 94) Copyright 2008 Corbett Life Science, a QIAGEN Company.

### Detection of intracellular HCV by flow cytometry

In this test Flow cytometry was used to evaluate the antiviral activity of the purified proteins (hLf, cLf, bLf and sLf). The HepG2 cells were suspended at 1.0 × 10^5^ cells/ml in DMEM culture media. The cells were infected with HCV-infected serum and incubated for 24 h at 37°C, 5% CO2 and 88% humidity. The purified Lf forms were added at concentrations of 0.25 and 0.5 mg/ml. Positive HepG2 (1.0 × 10^5^) cells were infected with HCV and negative HepG2 (1.0 × 10^5^) cells only without infection control cultures were included. The cells were incubated for four days at 37°C, 5% CO2 and 88% humidity. The cells were washed three times from debris and dead cells by using 1.0 × PBS. Intracellular labeling was performed by indirect immunofluorescence. Cells were centrifuged and supernatants were removed. Cell pellets were washed twice with 1.0 × PBS containing 1% normal goat serum (2% bovine serum albumin), cells were incubated with 4% paraformaldehyde for 10 min and 0.1% Triton X-100 in Tris buffer (pH 7.4) for 6 min. After washing three times with 1.0 × PBS, the cells were incubated with monoclonal antibody against HCV core (1:1,000) was added to the cell suspension and incubated at room temperature for 1 h [14,15,39]. Then, the cells were washed three times with 1.0 × PBS and the cells were immunofluorescence stained with fluorescein-conjugated goat anti-mouse and incubated at 4°C for 30 min. After being washed, the cells were suspended in 2 ml PBS and analyzed by Flow cytometry (Partic, Germany) [12,24].

### Statistical analysis

Most measurements were repeated three times and the results are presented as the mean plus standard deviation. Data were analyzed by using Student’s t-test.

## Abbreviations

HCV: Hepatitis C Virus; HBV: Hepatitis B Virus; HIV: Human Immunodeficiency Virus; HSV: Herpes Simplex Virus; PBMCs: Peripheral blood monocyte cells; RT-PCR: Reverse transcriptase-polymerase chain reaction; BSA: Bovine serum albumin; Lf: Lactoferrin; bLf: Bovine lactoferrin; cLf: Camel lactoferrin; hLf: Human lactoferrin; Lfs: Lactoferrins; PBS: phosphate buffer saline; MTT: Thiazolyl blue tetrazolium bromide; DMEM: Dulbecco's modefied eagle Medium; SDS-PAGE: Sodium dodecyl sulfate polyacrylamide gel; SEM: Standard Error of the mean.

## Competing interests

Authors declare that they have no competing interest.

## Authors’ contributions

EME, performed tissue culture, viral screening research, protein purifications and all immunoassays and wrote the draft of manuscript; LS, help in data organization, data analysis, manuscript revision; HAA, contributed new reagents/analytical tools and contributed in MS writing; and EMR, design research, data management, manuscript finalizing in its final form. All the authors read and approved the final manuscript.
